# Effect of Coenzyme Q10 Supplementation on Lipid and Glycaemic Profiles: An Umbrella Review

**DOI:** 10.3390/jcdd11120377

**Published:** 2024-11-25

**Authors:** Silvana Patiño-Cardona, Miriam Garrido-Miguel, Carlos Pascual-Morena, Carlos Berlanga-Macías, Maribel Lucerón-Lucas-Torres, Sofía Alfaro-González, Irene Martínez-García

**Affiliations:** 1 Health and Social Research Center, Universidad de Castilla-La Mancha, 16071 Cuenca, Spain; silvana.patino@alu.uclm.es (S.P.-C.); miriam.garrido@uclm.es (M.G.-M.); carlos.berlanga@uclm.es (C.B.-M.); mariaisabel.luceron@uclm.es (M.L.-L.-T.); sofia.alfaro@alu.uclm.es (S.A.-G.); 2 Faculty of Nursing, Universidad de Castilla-La Mancha, 02006 Albacete, Spain; 3 CarVasCare Research Group (2023-GRIN-34459), Facultad de Enfermería de Cuenca, Universidad de Castilla-La Mancha, 16001 Cuenca, Spain; irene.mgarcia@uclm.es

**Keywords:** nutraceutical, lipid profile, glycaemic profile, cardiovascular risk, systematic review, meta-analysis

## Abstract

Coenzyme Q10 (CoQ10) has been suggested as an adjunct therapy for endocrine and metabolic disorders. The aim of this study was to synthesise the evidence for the effect of CoQ10 supplementation on lipid and/or glycaemic alterations, including total cholesterol (TC), LDL- and HDL-cholesterol (LDL-C and HDL-C), lipoprotein a, fasting blood glucose (FBG), haemoglobin A1c (HbA1c), fasting insulin and Homeostatic Model Assessment of Insulin Resistance. A systematic search was conducted in Medline, Scopus, Web of Science and the Cochrane Library from their inception to July 2024. Meta-analyses that evaluated the effect of CoQ10 on the lipid or glycaemic profiles were included. Results were expressed as mean difference (MD) or standardised mean difference (SMD). CoQ10 showed an effect on the glycaemic profile, especially on FBG (MD from −11.21 to −5.2 mg/dL, SMD from −2.04 to −0.17) and on HbA1c (MD from −1.83 to −0.12%, SMD of −0.30). CoQ10 may also have an effect on the lipid profile, such as TC, triglycerides, HDL-C and even LDL-C, although the inconsistency of the results was somewhat higher. Supplementation with CoQ10 may be beneficial, especially in populations with diabetes mellitus or other endocrine and metabolic disorders. It could also have some effect on lipid parameters, which, together with the above, may reduce cardiovascular morbidity and mortality, although this is something that needs further research.

## 1. Introduction

There is currently a pandemic of biochemical and metabolic disorders that cause high morbidity and mortality, including dyslipidaemia and glycaemic disorders. Among the former, hypercholesterolaemia (total cholesterol or TC), elevated levels of low-density lipoprotein (LDL-C), low levels of high-density lipoprotein (HDL-C), hypertriglyceridemia and elevated levels of lipoprotein a [Lp(a)] stand out, while among the latter, elevated fasting plasma glucose (FPG), glycated haemoglobin (haemoglobin A1c), fasting insulin (FI) and Homeostatic Model Assessment for Insulin Resistance (HOMA-IR) stand out [1,2,3]. In general, it is estimated that up to 50% of the population in high-income countries have hypercholesterolaemia and about 10% have diabetes mellitus (DM), with type 2 DM, characterised by insulin resistance, being more common than type 1 DM, characterised by a deficiency in its endogenous synthesis [1,2,4]. These conditions increase the risk of neuropathy, retinopathy, atherosclerosis and cardiovascular disease [5,6].

Although there are genetic factors associated with these conditions, the main risk factors are unhealthy lifestyles, including excess weight, sedentary lifestyle, alcohol and drug abuse, kidney disease and high blood pressure, among others. In turn, the treatment of DM and dyslipidaemia generally begins with lifestyle changes, including increased physical activity, weight loss if the person has excess weight and a reduction in the intake of saturated fats and simple sugars. If lifestyle changes fail or are insufficient, pharmacological treatment is initiated, such as the use of statins, bile acid sequestrants or fibrates for dyslipidaemia and biguanides, sulfonylureas or insulin for DM [5,7].

In addition to pharmacological treatments, there are nutraceuticals that may have some beneficial effects when added to standard treatment. These include, for example, vitamin D, probiotics and prebiotics, magnesium, plant sterols and n-3 polyunsaturated fatty acids [8,9]. In addition, the use of coenzyme Q10 (CoQ10) has been proposed for both types of disorders. CoQ10 (ubiquinone in its oxidised form and ubiquinol in its reduced form) is a fat-soluble molecule that is essential to produce ATP, as it is part of the mitochondrial electronic chain, which is very important in cells with high energy requirements. It is mainly found in the cells of the heart, liver, kidneys and pancreas, although it is also detected in plasma, associated with cholesterol-transporting lipoproteins. In addition, CoQ10 may reduce oxidative stress associated with insulin resistance and pancreatic β-cell function, improving pancreatic and endothelial function and cellular metabolism [10,11,12].

Despite the potential effect of CoQ10 on these changes, the evidence to date is inconsistent. Therefore, the aim of this study was to synthesise the effect of CoQ10 on the lipid profile, i.e., TC, LDL-C, HDL-C and Lp(a), and on the glycaemic profile, i.e., FPG, haemoglobin A1c, FI and HOMA-IR.

## 2. Materials and Methods

An umbrella review of systematic reviews and meta-analyses was conducted in accordance with the PRISMA statement (Preferred Reporting Items for Systematic Reviews and Meta-Analyses) and the Cochrane Collaboration Handbook [13,14]. The protocol was previously registered in PROSPERO (CRD42024542621).

### 2.1. Search Strategy

A systematic search was conducted in the Medline (via PubMed), Scopus, Web of Science and Cochrane Library databases from their inception to July 2024. In addition, both the grey literature and the references of included studies were also reviewed. If the full text of the article was not available, attempts were made to contact the authors of the studies. The full search is described in Appendix S1.

Systematic search was conducted by two reviewers (S.P.-C. and C.P.-M.), and disagreements were resolved by consensus or by a third reviewer (I.M.-G.).

### 2.2. Inclusion/Exclusion Criteria

Systematic reviews and meta-analyses evaluating the effect of CoQ10 supplementation on lipid and glycaemic profiles were included. The PICO strategy was used to determine inclusion and exclusion criteria.

Inclusion criteria were as follows: (1) population—participants with pathologies that alter the lipid or glycaemic profile, such as DM, metabolic syndrome, cardiovascular disease, obesity and chronic kidney disease; (2) study design—systematic review with meta-analysis; (3) intervention—CoQ10 supplementation to standard treatment; (4) outcomes—TC, LDL-C, HDL-C, triglycerides, Lp(a), FPG, haemoglobin A1c, FI and HOMA-IR.

Exclusion criteria were as follows: (1) multicomponent interventions in which CoQ10 supplementation was not the main component (although meta-analyses in which some study included the combination of CoQ10 with another component were not excluded); (2) articles whose language was not English or Spanish.

Study selection was conducted by two reviewers (S.P.-C. and C.P.-M.), and disagreements were resolved by consensus or by a third reviewer (I.M.-G.).

### 2.3. Data Extraction

An ad hoc table was created with the following data extracted from the included studies: (1) reference (authors and year of publication); (2) year of studies included in meta-analysis (minimum–maximum); (3) number of studies included in each meta-analysis; (4) sample size of each meta-analysis; (5) age of participants in studies included in each meta-analysis (minimum–maximum); (6) daily dose of CoQ10 in milligrams (minimum–maximum); (7) duration of included interventions (minimum–maximum); (8) outcomes.

### 2.4. Risk of Bias Assessment

The AMSTAR-2 tool was used to assess the risk of bias [15]. This tool uses 16 items to rate each study. Depending on the domains involved and whether they are critical or not, each study can receive an overall rating from high to critically low quality.

Risk of bias assessment was conducted by two reviewers (S.P.-C. and C.P.-M.), and disagreements were resolved by consensus or by a third reviewer (I.M.-G.).

### 2.5. Quality of Evidence Assessment

The Grading of Recommendations, Assessment Development and Assessment (GRADE) tool was used to rate the quality of the evidence [16]. This tool rates the evidence for each intervention outcome as high, moderate, low or very low, considering a different set of factors.

### 2.6. Data Synthesis

In order to synthesise the data, an ad hoc table was created to summarise the results for each outcome and forest plots were used to present them graphically.

As the main objective of this review was to assess the efficacy of CoQ10 compared with no CoQ10, continuous variables were expressed as mean difference (MD) or standardised mean difference (SMD) and their confidence intervals (95%CI). The 95%CI was extracted directly or calculated from the standard error (SE) extracted from the trials or, in exceptional cases, from the *p*-value [17]. SMD effect sizes were considered small, medium or large if they were at or near 0.2, 0.5 and 0.8, respectively [18]. Heterogeneity (*I*^2^) from the original meta-analyses was included in the forest plots., and it was classified as not important (*I*^2^ < 30%), moderate (*I*^2^ = 30–50%), substantial (*I*^2^ = 50–75%) or considerable (*I*^2^ > 75%) [14]. Finally, subgroup studies were conducted, considering the dose of CoQ10 used, the length of the interventions, if another supplement is used with CoQ10, participants’ disease type if not a disease-only meta-analysis, type of CoQ10 (ubiquinone or ubiquinol), baseline FPG values and high-quality studies.

The statistical software Stata v15 (StataCorp, College Station, TX, USA) was used for the graphical presentation of the results.

## 3. Results

**Figure 1:**
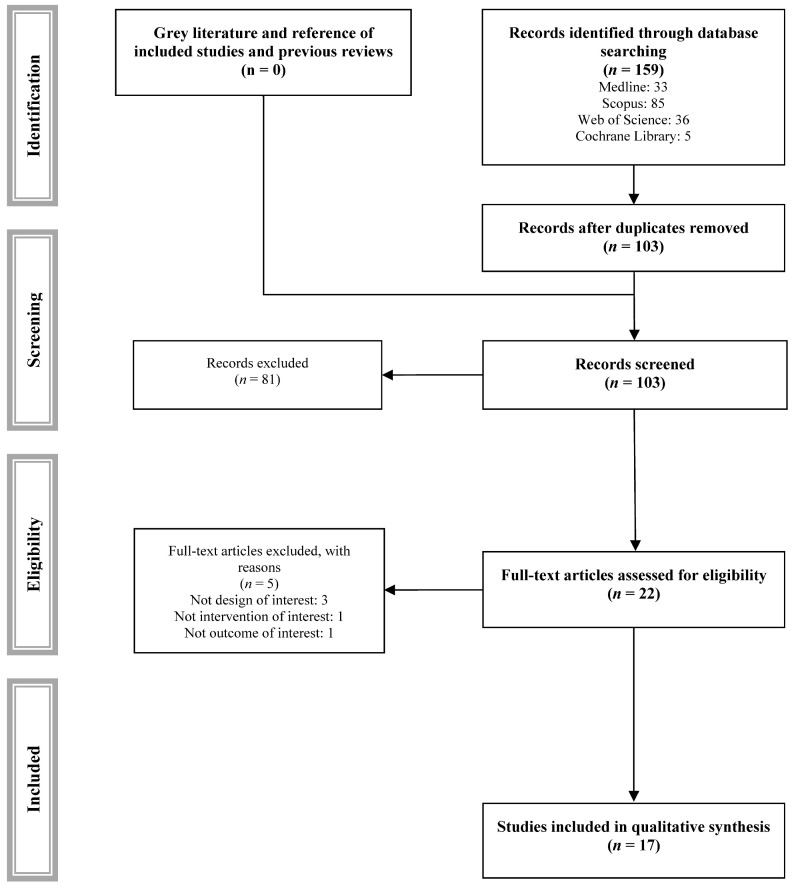
PRISMA study selection flowchart.

Of the one hundred and fifty-nine studies identified, twenty-two were considered eligible for inclusion and, after reading the full text, seventeen met the inclusion and exclusion criteria (Figure 1) [19,20,21,22,23,24,25,26,27,28,29,30,31,32,33,34,35], and five were excluded for justified reasons (Supplementary Table S1).

The included meta-analyses were published between 2015 and 2023, and the trials were conducted between 1959 and 2022. The participants were mostly adults, with the oldest being 89 years old. The intervention groups consisted of CoQ10 supplementation in addition to usual care, although some of the trials also included a combination with another substance (as the main compound studied, CoQ10). The daily doses of CoQ10 ranged from 20 to 1200 mg, and the duration of the interventions ranged from 2 weeks to 1 year. Finally, for lipid alterations, thirteen studies assessed the effect on TC, HDL-C, LDL-C and triglycerides, and two included Lp(a), while for glycaemic alterations, twelve studies assessed the effect on FPG, nine on haemoglobin A1c, eight on FI and seven on HOMA-IR. The characteristics of the included studies are described in Table 1, and the characteristics of the interventions are detailed in Supplementary Table S2.

### 3.1. Systematic Review

**Figure 2:**
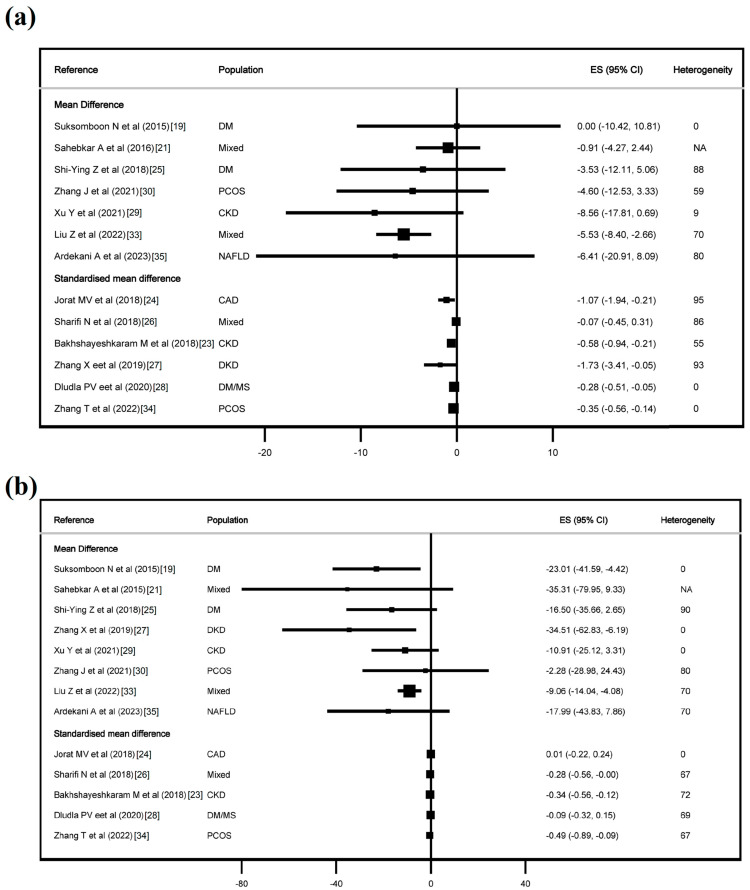
Effect of coenzyme Q10 on total cholesterol (**a**) and triglycerides (**b**). Refs. [19,21,23,24,25,26,27,28,29,30,33,34,35].

**Figure 3:**
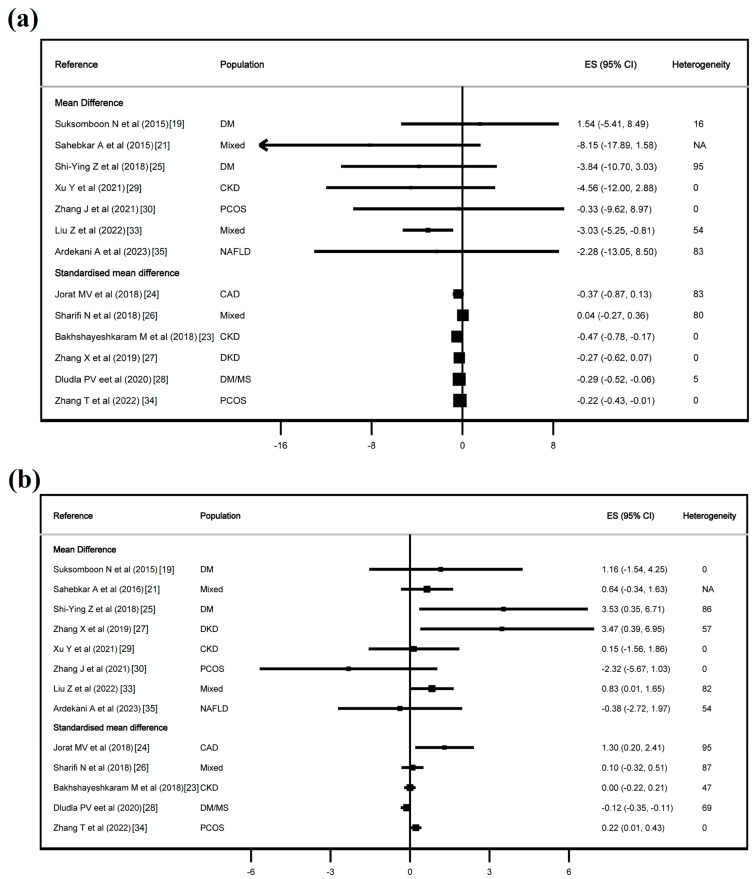
Effect of coenzyme Q10 on LDL-cholesterol (**a**) and HDL-cholesterol (**b**). Refs. [19,21,23,24,25,26,27,28,29,30,33,34,35].

**Figure 4:**
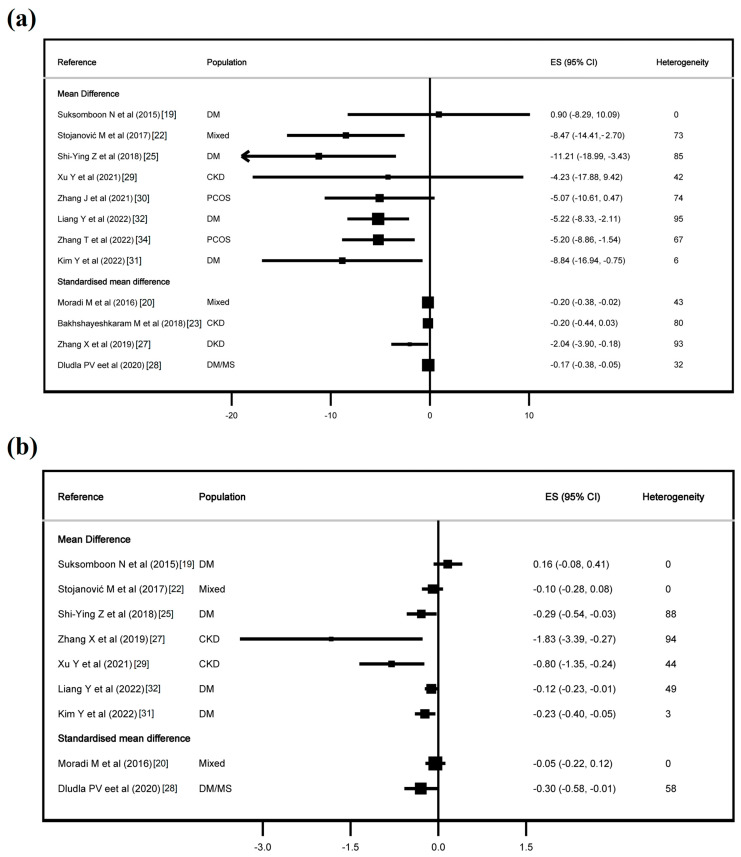
Effect of coenzyme Q10 on fasting plasma glucose (**a**) and haemoglobin A1c (**b**). Refs. [19,20,22,23,25,27,28,29,30,31,32,34].

**Figure 5:**
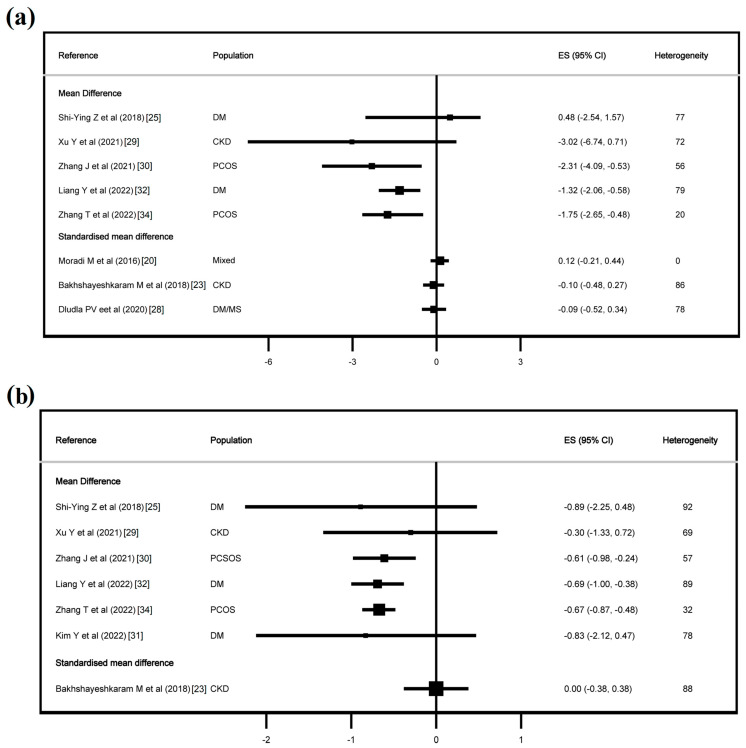
Effect of CoQ10 supplementation on fasting insulin (**a**) and HOMA-IR (**b**). Refs. [20,23,25,28,29,30,31,32,34].

Figure 2, Figure 3 and Figure S1 show the effect of CoQ10 on the lipid profile, and Figure 4 and Figure 5 show the effect of CoQ10 on the glycaemic profile.

In terms of lipid profile, CoQ10 supplementation showed an effect on TC in six of thirteen studies, with an MD of −5.53 mg/dL (−8.40, −2.55) and an SMD from −0.28 (−0.51, −0.06) to −1.73 (−3.41, −0.05). For LDL-C, CoQ10 had an effect in four of thirteen studies, with an MD of −3.03 mg/dL (−5.25, −0.81) and an SMD from −0.22 (−0.43, −0.01) to −0.47 (−0.78, −0.17). In HDL-C, CoQ10 had an effect in five of thirteen studies, with an MD from 0.83 mg/dL (0.01, 1.65) to 3.53 mg/dL (0.35, 6.71), and an SMD from 0.22 (0.01, 0.43) to 1.30 (0.20, 2.41). For triglycerides, CoQ10 had an effect in six of thirteen studies with an MD from −9.06 mg/dL (−14.04, −4.08) to −34.51 mg/dL (−62.83, −6.19), and an SMD from −0.28 (−0.56, −0.00) to −0.49 (−0.89, −0.09). Finally, in Lp(a), CoQ10 had an effect in one of two studies, with an SMD of −3.55 (−5.50, −1.58).

With regard to the glycaemic profile, CoQ10 supplementation had an effect on FPG in eight of twelve studies and on glycaemic haemoglobin in six of nine studies, the former with an MD from −11.21 mg/dL (−18.99, −3.43) to −5.20 mg/dL (−8.86, −1.54) and an SMD from −2.04 (−3.90, −0.18) to −0.17 (−0.38, −0.05), and for haemoglobin A1c, an MD from −1.83% (−3.39, −0.27) to −0.12% (−0.23, −0.01) and an SMD of −0.30 (−0.58, −0.01). Finally, CoQ10 had an effect on FI in three of eight studies, with an MD from −2.31 (−4.09, −0.53) to −1.32 (−2.06, −0.58), and on HOMA-IR in three of seven studies, with an MD from −0.69 (−1.00, −0.38) to −0.61 (−0.98, −0.24).

Subgroup analyses have shown inconsistent results. However, it is suggested that the optimal dose of CoQ10, especially in the glycaemic profile, is less than 150–200 mg/day. In addition, although some meta-analyses did not show this trend, in general there was a trend in the most recent meta-analyses towards a greater effect when the interventions were longer. Similarly, in the TC, there was a favourable trend towards a larger effect with CoQ10 supplementation without other nutraceuticals, as with FPG. Finally, the results by disease type and CoQ10 type were inconsistent. However, higher basal FPG levels were associated with greater FPG reductions. In addition, the two meta-analyses considered as higher quality according to the AMSTAR-2 criteria showed a statistically significant effect on triglycerides, FPG and glycaemic haemoglobin (Supplementary Table S3).

The heterogeneity was generally substantial to considerable, except in some meta-analyses where it was less than 30%.

### 3.2. Risk of Bias Assessment

According to the risk of bias assessment using the AMSTAR-2 tool, two out of seventeen studies (11.8%) were of high quality, six (35.3%) were of low quality and nine (52.9%) were of very low quality. The domains most affected were failure to report that the protocol/methodology was conducted prior to the study, failure to correctly describe the search strategy, failure to include a list of excluded studies with justification, failure to report the funding of included studies, and failure to assess the risk of bias of included studies (Supplementary Table S4).

### 3.3. Quality of Evidence Assessment

According to the GRADE tool, the effect of CoQ10 supplementation on haemoglobin A1c and FPG had high and moderate quality of evidence, respectively, while the rest had low or very low quality of evidence (Supplementary Table S5).

## 4. Discussion

To our knowledge, this is the first overview of systematic reviews and meta-analyses conducted to date. Our results show that CoQ10 could have a positive effect on FPG and haemoglobin A1c. Specifically, supplementation with CoQ10 could reduce FPG by approximately 5.00 to 11.00 mg/dL, and haemoglobin A1c by approximately 0.12 to 1.83%, although the initial meta-analyses conducted did not reach these results. For other parameters, the data are less consistent, although this does not rule out the possibility that supplementation with CoQ10 could have some benefit on the lipid profile, such as triglycerides and other lipid parameters.

The data show that CoQ10 could have a significant effect mainly on glycaemic parameters, especially FPG and haemoglobin A1c. This finding was supported by subgroup analyses, which showed a greater reduction in FPG when baseline levels were higher. However, some discrepancies were observed. There are several factors which could respond to the findings, including the characteristics and age of the participants, the dose and duration of treatment and the sample size. Firstly, those that showed a beneficial effect tended to include participants with a metabolic disease such as DM. This could be due to the CoQ10 deficiency usually observed in DM2, in addition to the oxidative stress and mitochondrial diffusion associated with the disease, which could be improved by the antioxidant and metabolic effects of CoQ10 [36]. Regarding the age, endogenous CoQ10 biosynthesis declines with age. This is detrimental because of the role of CoQ10 in the mitochondrial electron chain, which is essential for energy production, and its redox and antioxidant capacity, which prevents ferroptosis induced by oxidative damage. Thus, a greater effect can be observed in the elderly population, although there are some exceptions that require further research [37]. In terms of efficacy, the optimal dose is thought to be in the region of 100–200 mg/day, without a completely linear relationship between dose and absorption/plasma concentrations [32]. Stojanovic et al. suggested that doses of less than 200 mg/day and duration of less than 12 weeks may be beneficial for lowering blood glucose, while higher doses may be toxic to β-cells [22,38]. This is in line with Moradi et al. [20], who showed that 120 mg/day for 8 weeks reduced oxidative stress, which is greater in people with DM and is directly related to hyperglycaemia and insulin resistance. Finally, the studies with small sample sizes did not show improvements in haemoglobin A1c or FPG after supplementation with CoQ10. This is not surprising for a non-pharmacological supplement, where the effect it could have is relatively limited compared to other DM-specific drugs.

For the lipid profile, the results were contradictory, although the fact that it showed an effect in some trials does not rule out that it has a real effect in some circumstances or for some parameters, such as total cholesterol or triglycerides. It is interesting to note that the meta-analysis with the largest sample size showed a modest improvement in the four lipid parameters, i.e., total cholesterol, LDL-C, HDL-C and triglycerides, with the effect in some cases depending on the baseline population and the dose. Finally, Lp(a) was reduced in one of the two included meta-analyses. It has been suggested that the effect on Lp(a) is dependent on its basal level, being more effective when the basal level of Lp(a) is higher [21]. Similarly, doses of less than 150 mg/day seem to be more effective, as does a duration of less than 8 weeks [24]. There are several reasons for this unexpected finding, which are not mutually exclusive. First, a limited number of trials were included in each subgroup, namely one to four trials, which limits the statistical power of the meta-analysis. Furthermore, if only one study was included, it is not really a meta-analysis. Second, it has been observed that the absorption efficiency of chronically administered CoQ10 decreases, which may affect its effect [39]. Thirdly, the negative association between the dose of CoQ10 and the observed effect may be spurious because, in some cases, the trials with participants with lower baseline Lp(a) were supplemented with higher doses of CoQ10 [21]. Therefore, their margin for improvement is smaller. In any case, these results show that although CoQ10 may have some beneficial effects on the lipid profile, the factors that make it more effective are largely unknown.

In addition to the above, the effects observed on the glycaemic profile can be explained by several mechanisms of action of CoQ10. The first is the oxidative effect of CoQ10 on glycolysis. In people with DM, the enzyme glycerol-3-phosphate dehydrogenase (G3PD), which plays an important role in glucose metabolism, is altered, leading to impaired insulin production. CoQ10, through its effect on oxidative glycolysis, is able to act in the absence of G3PD activity and increase insulin sensitivity [40]. The second mechanism of action is CoQ10’s activity in the mitochondrial respiratory chain [12], which leads to an increased demand for pyruvate and an increase in cellular glucose uptake, lowering blood glucose levels. Another mechanism to highlight is the role of CoQ10 in inducing the genetic expression of nuclear receptors PPARs (Peroxisomal Proliferator Activated Receptors). These receptors bind to a range of ligands and regulate the expression of genes involved in lipid and glucose metabolism. PPARs have a hypoglycaemic effect by sensitising peripheral tissues to the action of insulin [41]. As the glitazones used in DM also act at this level, an increase in their effect could be hypothesised.

The results obtained suggest a significant effect on the glycaemic profile, with a greater effect on basal glucose and glycaemic haemoglobin, so supplementation with CoQ10 could be of interest in people with glycaemic disorders such as type 2 DM or other associated metabolic syndromes, such as chronic kidney disease or polycystic ovary syndrome, always under medical supervision. At the same time, it is important to highlight the inconsistency of the data, since we found studies with an effect on the lipid profile and studies without an effect, which would lead us to the need for further research to know the optimal supplementation conditions in which supplementation is effective, such as dose, type of pathology and age, among others.

It is worth considering the effect and possible benefits of CoQ10 supplementation in reducing cardiovascular disease, as it is known that small reductions in LDL-C or haemoglobin A1c have an important effect on cardiovascular morbidity and mortality at a population level [33,42].

Interestingly, in addition to a possible effect on cardiovascular risk factors (i.e., on the lipid and glycaemic profiles), other potential effects need to be considered, such as the effect on blood pressure levels, particularly systolic blood pressure, in different populations. It may also be beneficial in heart failure, reducing morbidity and mortality [43,44,45,46]. It is therefore necessary to study the clinical use of CoQ10 in the management of these diseases, which could improve the quality of life of these patients, without forgetting the importance of its additive or synergistic use with drugs prescribed for different pathologies (oral antidiabetics and statins, among others). It is also very important to resolve inconsistencies and to be able to answer why it shows an effect in some studies and not in others.

Finally, in order to contextualise the results of this study, previous reports showed that in DM2, the most commonly used drug, metformin, had an effect on FPG and haemoglobin A1c of around −31 mg/dL and −1.3%, respectively [47]. Although the effect of CoQ10 is less than that of oral antidiabetic drugs, it should be noted that the use of CoQ10 is never considered as a monotherapy, but rather as an adjunct to usual treatment. Therefore, it is expected to have an additive or synergistic effect on the effect of other oral antidiabetic drugs and may provide additional benefit in the management of glycaemia.

One of the aspects that needs to be investigated in the future is the effect of the different forms of CoQ10 (i.e., ubiquinone and ubiquinol). In the included meta-analyses, the type of CoQ10 used was rarely reported, but it is likely that most trials used ubiquinone. Ubiquinol could have a higher bioavailability than ubiquinone, which could lead to a greater effect, although this is controversial and not yet demonstrated [48]. The subgroup analysis of this review showed no effect of ubiquinol, probably because only two trials were included. In addition, more research is needed into the effects of COQ10 in combination with other supplements. In subgroup analyses, some studies did not reach statistical significance: one study showed a favourable trend when COQ10 was used alone, while another showed the opposite. Conversely, in one study that did reach statistical significance, especially for total cholesterol, the trend was favourable when COQ10 was used alone. These contradictory results are probably due to the impossibility of controlling several variables or strata at the same time, which requires standardisation in future trials and especially in meta-analyses, with more concrete populations, more standardised interventions and standardised doses, among others.

### Limitations

Some limitations of this study should be considered. First, all the included systematic reviews aimed to assess the effect of CoQ10, but some of the trials included in these reviews may have been multicomponent, which could not always be assessed in the subgroup study. In addition, these types of trials were not always well detailed. Some trials only compared CoQ10 with placebo, without taking into account the usual treatment that these people were most likely to be taking. When they were reported, they sometimes did not include the dose of the usual treatment. Although the trials generally compared the addition of CoQ10 with a placebo, the use of other medications could potentially affect the observed effect of CoQ10. Finally, systematic review authors did not include information about the schedule of the intervention and control groups. Second, the effect of CoQ10 on plasma levels was not generally assessed, nor was whether participants were CoQ10 deficient at the start of the trials, which limits the interpretation of the results at a physiological level. Third, the effect of age on the effect of CoQ10 was not studied, so studies specifically looking at this factor are needed. Fourth, the quality of more than half of the included studies is low or very low. Although the risk of bias or the quality of the studies does not invalidate their results, they should be considered with some caution. Fifth, and related to the above, some studies did not assess the risk of bias or did not discuss it, so it was not possible to assess the possible effect of the risk of bias on the results obtained. Sixth, different techniques were used to measure the different glycaemic and lipid parameters, which could affect the precision of the results because they were not measured with the same technique. Seventh, the interventions in each meta-analysis varied in terms of participants, duration and CoQ10 dose. Although an attempt was made to control for this, trials with more homogeneous participants and with dose and duration escalation are needed. Eighth, most of the studies used ubiquinone, so the available evidence is almost exclusively limited to this form of CoQ10. Only one study explicitly reported on the use of ubiquinol, without finding a statistically significant effect, probably due to the small number of included trials.

## 5. Conclusions

The available evidence shows that CoQ10 could improve the glycaemic and lipid profiles in the population that has an alteration in these parameters, especially the glycaemic profile, probably in the population with DM, although further research is needed to increase the consistency of the data and thus be able to elucidate the conditions under which CoQ10 supplementation has an optimal effect, i.e., at what age, in what diseases and at what doses it provides greater benefit, among other variables. However, an effect on the lipid profile cannot be ruled out, which, together with the likely improvement in the glycaemic profile, could lead to a reduction in cardiovascular risk.

## Figures and Tables

**Table 1:** Characteristics of the included studies.

Reference	Year	№ Studies	Population	N	Age	Dose (mg)	Length (weeks)	Outcome								
								TC	HDL	LDL	TG	LPA	FB	Hb	FI	HIR
Suksomboon N et al. (2015) [19]	1999–2013	7	Diabetes Mellitus	356	NA	100–200	12–24	✓	✓	✓	✓	-	✓	✓	-	-
Moradi M et al. (2016) [20]	1999–2014	16	Various diseases	780	NA	100–600	4–25	-	-	-	-	-	✓	✓	✓	-
Sahebkar A et al. (2016) [21]	1999–2014	6	Various diseases	409	42.5–70.1	100–300	4–12	✓	✓	✓	✓	✓	-	-	-	-
Stojanovié M et al. (2017) [22]	1997–2015	18	Various diseases	930	35.2–68.9	100–300	4–24	-	-	-	-	-	✓	✓	-	-
Bakhshayeshkaram M et al. (2018) [23]	2000–2017	7	Coronary artery disease	384	44.2–66.3	30–200	8–12	✓	✓	✓	✓	-	✓	-	✓	✓
Jorat MV et al. (2018) [24]	1999–2017	8	Coronary artery disease	526	47.6–68.9	100–300	4–48	✓	✓	✓	✓	✓	-	-	-	-
Shi-Ying Z et al. (2018) [25]	2002–2008	13	Type 2 DM	765	45.2–65.0	100–200	8–24	✓	✓	✓	✓	-	✓	✓	✓	✓
Sharifi N et al. (2018) [26]	1999–2016	21	Various diseases	1039	18.0–75.0	100–300	4–48	✓	✓	✓	✓	-	-	-	-	-
Zhang X et al. (2019) [27]	2011–2018	5	Diabetic kidney disease	175	18.0–83.0	30–1000	10–12	✓	✓	✓	✓	-	✓	✓	-	-
Dludla PV et al. (2020) [28]	2015–2018	12	Diabetes Mellitus and Metabolic Syndrome	650	46.2–62.9	20–400	8–24	✓	✓	✓	✓	-	✓	✓	✓	-
Xu Y et al. (2021) [29]	1983–2018	12	Chronic kidney disease	542	40.0–85.0	30–1200	4–24	✓	✓	✓	✓	-	✓	✓	✓	✓
Zhang J et al. (2021) [30]	2017	2	Polycystic ovary syndrome	100	24.5–25.3	100	12	✓	✓	✓	✓	-	✓	-	✓	✓
Kim Y et al. (2022) [31]	1999–2019	11	Type II DM	524	45.2–68.0	100–200	8–24	-	-	-	-	-	✓	✓	-	✓
Liang Y et al. (2022) [32]	1994–2020	40	Various diseases	2424	19.9–70.1	100–900	4–24	-	-	-	-	-	✓	✓	✓	✓
Liu Z et al. (2022) [33]	1994–2022	50	Various diseases	2794	19.0–68.0	100–900	2–52	✓	✓	✓	✓	-	-	-	-	-
Zhang T et al. (2022) [34]	2014–2021	9	Polycystic ovary syndrome	1021	24.7–30.4	100–200	8–12	✓	✓	✓	✓	-	✓	-	✓	✓
Ardekani A et al. (2023) [35]	2014–2020	6	Non-alcoholic fatty liver	300	NA	20–100	4–12	✓	✓	✓	✓	-	-	-	-	-

Abbreviations: NA—Not applicable/available; TC—Total cholesterol; HDL—HDL-C; LDL—LDL-C; TG—Triglycerides; LPA—Lipoprotein a; FB—Fasting blood glucose; Hb—Glycated haemoglobin; FI—Fasting insulin; HIR—HOMA-IR; ✓—Outcome included.

## Data Availability

Data are available upon reasonable request to the corresponding author.

## Supplementary Materials

The following supporting information can be downloaded at: https://www.mdpi.com/article/10.3390/jcdd11120377/s1, Table S1: Excluded studies with justified reasons; Table S2: Characteristics of the intervention and control groups; Table S3: Subgroup studies; Table S4: Risk of bias assessment; Table S5: Quality of evidence assessment; Figure S1: Effect of CoQ10 supplementation on Lp(a); Appendix S1: Search strategy. [49,50,51,52,53] are cited in the Supplementary Materials.
