# Supplementary material for: Effect of Coenzyme Q10 Supplementation on Lipid and Glycaemic Profiles: An Umbrella Review
Source: J Cardiovasc Dev Dis. 2024 Nov 25;11(12):377. doi: 10.3390/jcdd11120377 (PMC11678712; doi:10.3390/jcdd11120377)
Supplement: Supplementary file 1 [file jcdd-11-00377-s001.zip › jcdd-3275032-supplementary.pdf]

SUPPLEMENTARY MATERIAL

Effect of Coenzyme Q10 supplementation on lipid and glycaemic profile: an umbrella review

Journal of Cardiovascular Development and Disease

INDEX

Table S1. Excluded studies with justified reasons.

Table S2. Characteristics of the intervention and control groups.

Table S3. Subgroup studies.

Table S4. Risk of bias assessment.

Table S5. Quality of evidence assessment.

Figure S1. Effect of CoQ10 supplementation on Lp(a).

Appendix S1. Search strategy.

Table S1. Excluded studies with justified reasons.

Reference	Main reason
Ayers J et al (2018) [1]	Not design of interest
Cogorno L et al (2023) [2]	Not design of interest
Chagan L et al (2002) [3]	Not design of interest
Pirro M et al (2016) [4]	Not intervention of interest
Aslani Z et al (2018) [5]	Not outcome of interest

Supplementary References

1. Ayers, J.; Cook, J.; Koenig, R.A.; Sisson, E.M.; Dixon, D.L. Recent Developments in the Role of Coenzyme Q10 for Coronary Heart Disease: A Systematic Review. *Curr. Atheroscler. Rep*. **2018**, *20*, 29.

2. Cogorno, L.; Formisano, E.; Vignati, A.; Prigione, A.; Tramacere, A.; Borgarelli, C.; Sukkar, S.G.; Pisciotta, L. Non-alcoholic fatty liver disease: Dietary and nutraceutical approaches. *Liver Res*. **2023**, *7*, 216–227.

3. Chagan, L.; Ioselovich, A.; Asherova, L.; Cheng, J. Use of alternative pharmacotherapy in management of cardiovascular diseases. *Am. J. Manag. Care* **2002**, *8*, 270–285.

4. Pirro, M.; Mannarino, M.R.; Bianconi, V.; Simental-Mendía, L.E.; Bagaglia, F.; Mannarino, E.; Sahebkar, A. The effects of a nutraceutical combination on plasma lipids and glucose: A systematic review and meta-analysis of randomized controlled trials. *Pharmacol. Res*. **2016**, *110*, 76–88.

5. Aslani, Z.; Shab-Bidar, S.; Fatahi, S.; Djafarian, K. Effect of Coenzyme Q10 Supplementation on Serum of High Sensitivity C-Reactive Protein Level in Patients with Cardiovascular Diseases: A Systematic Review and Meta-Analysis of Randomized Controlled Trials. *Int J Prev Med* **2018**, *9*, doi:10.4103/ijpvm.IJPVM_263_17.

Table S2. Characteristics of the intervention and control groups.

Reference	Disease	Intervention	Type CoQ10	Comparator
Suksomboon N et al (2015)	Diabetes mellitus	CoQ10 + Usual treatment (Insulin, other antidiabetic drugs, antihypertensive drugs, lipid-lowering drugs, etc.)	Not specified	Placebo + Usual treatment
Moradi M et al (2016)	Various diseases	CoQ10 + Other drugs	Not specified	Placebo + Other drugs
Sahebkar A et al (2016)	Various diseases	CoQ10 or CoQ10 + Other drugs/supplements	Not specified	Placebo
Stojanovié M et al (2017)	Various diseases	CoQ10 + Usual treatment (not specified)	Not specified	Placebo + Usual treatment (not specified)
Bakhshayeshkaram M et al (2018)	Coronary artery disease	CoQ10 + Usual treatment (not specified). One study: CoQ10 + other nutraceuticals	Not specified	Placebo + Usual treatment (not specified)
Jorat MV et al (2018)	Coronary artery disease	CoQ10 + Usual treatment (not specified). One study: CoQ10 + other nutraceuticals	Not specified	Placebo + Usual treatment (not specified)
Shi-Ying Z et al (2018)	Type 2 DM	CoQ10 + Usual treatment or CoQ10 + fenofibrate + Usual treatment (not specified)	Not specified	Placebo + Usual treatment or Placebo + fenofibrate + Usual treatment (not specified)
Sharifi N et al (2018)	Various diseases	CoQ10 + Usual treatment (not specified)	Not specified	Placebo + Usual treatment (not specified)
Zhang X et al (2019)	Diabetic kidney disease	CoQ10 or CoQ10 + Usual treatment (Insulin, atorvastatin)	Not specified	Placebo or Placebo + Usual treatment (Insulin, atorvastatin)
Dludla PV et al (2020)	Diabetes Mellitus and Metabolic Syndrome	CoQ10 + Usual treatment (not specified). One study: CoQ10 + vitamin E	Not specified	Placebo + Usual treatment (not specified)
Xu Y et al (2021)	Chronic kidney disease	CoQ10 + Usual treatment (not specified)	Not specified	Placebo + Usual treatment (not specified)
Zhang J et al (2021)	Polycystic ovary syndrome	CoQ10 or CoQ10 + Vitamin E	Not specified	Placebo or Placebo + Vitamin E

Kim Y et al (2022)	Type II DM	CoQ10 + insulin/other antidiabetic drugs	Not specified	Placebo + insulin/other antidiabetic drugs
Liang Y et al (2022)	Various diseases	CoQ10	All ubiquinone except one study	Placebo or simvastatin/fenofibrate/ omega-3/vitamin E
Liu Z et al (2022)	Various diseases	CoQ10 or CoQ10 + Usual treatment (atorvastatin, simvastatin, fenofibrate, exercise, tocopherol, omega-3, carnitine)	All ubiquinone except two studies	Placebo or Placebo + Usual treatment (atorvastatin, simvastatin, fenofibrate, exercise, tocopherol, omega-3, carnitine))
Zhang T et al (2022)	Polycystic ovary syndrome	CoQ10 or CoQ10 + Usual treatment (metformin, vitamin E, clomiphene, omega-3)	Not specified	Placebo or Placebo + Usual treatment (metformin, vitamin E, clomiphene, omega-3)
Aldekani A et al (2023)	Non-alcoholic fatty liver	CoQ10 + Usual treatment (not specified) or CoQ10 + Usual treatment + other nutraceuticals	Not specified	Placebo + Usual treatment (not specified) or Placebo + Usual treatment + other nutraceuticals

Table S3. Subgroup studies.

- Effect of CoQ10 on lipid profile.

**Reference**	**Subgroup**	**TC**	**LDL-C**	**HDL-C**	**TG**	**LPA**
**By dosage**						
Sahebkar A et al (2016)	< 150 mg	-	-	-	-	-9.24 (-15.19, -.3.29)
	> 150 mg	-	-	-	-	-2.75 (-4.28, -1.23)
Bakhshayeshkaram M.et.al (2018)	< 200 mg	-1.05 (-1.68, -0.43)	-	-	-0.97 (-1.81, -0.13)	-
	> 200 mg	-0.28 (-0.52, -0.05)	-	-	-0.09 (-0.59, 0.40)	-
Jorat MV et al (2018)	< 150 mg	-1.53 (-2.83, -0.23)	-0.24 (-0.64, 0.16)	0.66 (0.03, 1.30)	-	-1.79 (-4.98, 1.40)
	> 150 mg	-0.52 (-1.78, 0.73)	-0.53 (-1.64, 0.57)	2.58 (-2.65, 7.81)	-	0.13 (-0.39, 0.66)
Liu Z et al (2022)	< 200 mg	-2.55 (-5.79, 0.70)	-3.45 (-7.03, 0.13)	0.87 (−0.60, 2.34)	−10.11 (−18.78, −1.43)	-
	> 200 mg	-2.30 (-11.12, -3.23)	-2.81 (-5.90, 0.29)	0.82 (−0.21, 1.86)	−8.37 (−14.79, −1.94)	-
Ardekani A et al (2023)	< 100 mg	−17.17 (−30.76, −3.57)	−6.43 (−24.32, 11.45)	0.12 (−4.48, 4.72)	−28.31 (−77.90, 21.27)	-

	> 100 mg	3.89 (−5.72, 13.51)	2.73 (−4.77, 10.24)	−1.22 (−3.70, 1.24)	−6.12 (−23.40, 11.14)	-
**By length**						
Sahebkar A et al (2016)	< 8 weeks	-	-	-	-	-3.14 (-4.92, -1.36)
	> 8 weeks	-	-	-	-	-11.72 (-21.01, 2.42)
Bakhshayeshkaram M.et.al (2018)	< 12 weeks	-0.70 (-1.46, 0.06)	-	-	-0.68 (-1.32, -0.04)	-
	> 12 weeks	-0.53 (-0.92, -0.13)	-	-	0.03 (-0.81, 0.87)	-
Jorat MV et al (2018)	< 8 weeks	0.06 (-0.26, 0.39)	-0.12 (-0.55, 0.30)	0.24 (-0.30, 0.78)	-	-1.64 (-5.14, 1.87)
	> 8 weeks	-1.94 (3.33, -0.54)	-0.62 (-1.55, 0.31)	3.14 (-0.96, 7.24)	-	-0.18 (-0.69, 0.32)
Sharifi N et al (2018)	< 12 weeks	-0.11 (-1.04, 0.81)	-0.06 (-0.87, 0.74)	-0.24 (-1.19, 0.71)	-0.51 (-1.14, 0.13)	-
	> 12 weeks	-0.05 (-0.34, 0.25)	0.11 (-0.08, 0.30)	0.27 (-0.14, 0.67)	-0.17 (-0.44, 0.11)	-
Liu Z et al (2022)	< 12 weeks	-7.95 (-11.99, -3.91)	−2.40 (−4.30, −0.50)	0.15 (−0.81, 1.11)	−6.36 (−15.02, 2.30)	-
	> 12 weeks	-5.53 (-8.40, -2.66)	−2.74 (−5.91, 0.43)	1.28 (−0.13, 2.68)	−10.50 (−16.76, −4.24)	-
Ardekani A et al (2023)	4 weeks	−0.2 (−13.31, 12.91)	1.2 (−10.46, 12.86)	−0.2 (−4.43, 4.03)	−0.8 (−22.59, 20.99)	-
	12 weeks	−8.33 (−27.3, 10.63)	−3.19 (−16.75, 10.35)	−0.45 (−3.51, 2.59)	−25.25 (−55.35, 4.84)	-
**Type of intervention**						
Bakhshayeshkaram M et al (2018)	With other supplements	-0.39 (-0.81, 0.02)	-	-	-0.41 (-1.24, 0.43)	-
	Without other supplements	-0.75 (-1.33, -0.18)	-	-	-0.30 (-1.00, 0.39)	-
Jorat MV et al (2018)	With other supplements	−4.50 (−13.39, 4.39)	-	-	-	-
	Without other supplements	−0.32 (−0.85, 0.22)	−0.37 (− 0.87, 0.13)	1.30 (0.20, 2.41)	-	−1.12 (−2.84, 0.61)
Liu Z et al (2022)	With other supplements	−2.69 (−7.64, 2.27)	−3.78 (−7.28, −0.28)	1.05 (−0.22, 2.32)	−8.36 (−19.20, 2.49)	-
	Without other supplements	−6.40 (−9.73, −3.07)	−2.93 (−5.59, −0.26)	0.72 (−0.36, 1.79)	−9.30 (−15.08, −3.53)	-
**Basal covariates**						
Sharifi N et al (2018)	With diabetes	-0.34 (-1.07, 0.39)	-0.20 (-0.74, 0.34)	-0.37 (-1.17, 0.42)	-0.72 (-1.38, -0.07)	-
	Without diabetes	0.08 (-0.39, 0.54)	0.23 (-0.16, 0.61)	0.42 (0.00, 0.82)	-0.01 (-0.03, 0.12)	-
Liu Z et al (2022)	Healthy	−12.30 (−14.89, −9.71)	−5.00 (−15.55, 5.55)	0.16 (−2.42, 2.73)	−6.47 (−29.99, 17.05)	-
	Diabetes	−2.08 (−6.18, 2.03)	0.00 (−4.18, −4.19)	0.17 (−1.37, 1.71)	−7.40 (−13.96, −0.84)	-
	Dyslipidaemia	−4.53 (−15.96, 6.89)	−2.78 (−9.11, 3.56)	3.69 (0.09, 7.30)	−9.81 (−26.67, 7.06)	-
	CVD	−0.98 (−3.66, 1.71)	−1.34 (−3.50, 0.82)	0.91 (−1.10, 2.91)	−4.76 (−13.48, 3.96)	-
	CKD	−11.72 (−26.88, 3.44)	−5.81 (−10.09, −1.53)	−2.69 (−4.82, −0.56)	−8.68 (−14.05, 8.68)	-
	Other diseases	−6.51 (−13.04, 0.02)	−9.19 (−15.14, −3.24)	0.73 (−0.32, 1.78)	−8.57 (−16.47, −0.66)	-
	Ubiquinone	−5.68 (−8.61, −2.75)	−3.02 (−5.32, −0.72)	0.77 (−0.05, 1.60)	−9.10 (−14.08, −4.11)	-
	Ubiquinol	0.47 (−13.81, 14.75)	−3.40 (−12.91, 6.11)	4.74 (−1.81, 11.30)	8.36 (−88.89, 105.61)	-
**High quality**						
Sharifi N et al (2018)	-	-0.07 (-0.45, 0.31)	0.04 (-0.27, -0.36)	0.10 (-0.32, 0.51)	-0.28 (-0.56, -0.00)	-

- Effect of CoQ10 on glycaemic profile.

Reference	Subgroup	FPG	HA1C	FI	HOMA-IR
**Dosage**					
Moradi M et al (2016	< 200 mg	-0.36 (-0.67, -0.05)	-0.04 (-0.35, 0.26)	-	-
	>200 mg	-0.13 (-0.34, 0.08)	-0.06 (0.27, 0.15)	-	-
Stojanović M et al (2017)	< 200 mg	-0.84 (-1.37, -0.31)	-	-	-
	>200 mg	-0.01 (-0.32, 0.29)	-	-	-
Bakhshayeshkaram M.et.al (2018)	< 200 mg	-0.45 (-0.71, -0.18)	-	-0.98 (-1.57, -0.40)	-0.94 (-1.52, 0.35)
	> 200 mg	0.62 (0.13, 1.11)	-	0.49 (0.01, 0.97)	0.66 (0.17, 1.15)
Liang Y et al (2022)	< 200 mg	-13.21 (-18.43, -7.98)	-0.47 (-0.83, -0.12)	-1.71 (-2.57, -0.85)	-0.97 (-1.44, -0.50)
	> 200 mg	-0.71 (-3.42, 1.99)	-0.03 (-0.15, 0.10)	-0.43 (-2.12, 1.27)	-0.54 (-1.17, 0.10)
**Length**					
Moradi M et al (2016	< 20 weeks	-0.22 (-0.41, -0.04)	-0.08 (-0.28, 0.12)	-	-
	> 20 weeks	-0.03 (-0.54, 0.48)	0.02 (-0.32, 0.37)	-	-
Stojanović M et al (2017)	< 12 weeks	-0.60 (-1.07, -0.12)	-	-	-
	> 12 weeks	-0.14 (-0.49, 0.20)	-	-	-
Bakhshayeshkaram M.et.al (2018)	< 12 weeks	0.23 (-0.56, 1.02)	-	0.49 (0.01, 0.97)	0.66 (0.17, 1.15=)
	> 12 weeks	-0.64 (1.21, -0.07)	-	-0.98 (-1.57, -0.40)	-0.94 (-1.52, -0.35)
Liang Y et al (2022)	< 12 weeks	-2.41 (-6.87, 2.06)	-0.06 (-0.28, 0.16)	-0.59 (-1.59, 0.42)	-0.24 (-0.52, 0.05)
	> 12 weeks	-7.59 (-11.66, -3.52)	-0.14 (-0.27, 0.00)	-1.51 (-2.52, -0.50)	-1.03 (-1.40, -0.65)
**Type of intervention**					
Moradi M et al (2016	With other supplements	0.08 (-0.30, 0.47)	-	-	-
	Without other supplements	-0.27 (-0.47, -0.08)	-	-	-
Bakhshayeshkaram M et al (2018)	With other supplements	-0.01 (-0.87, 0.84)	-	0.44 (-0.25, 1.13)	0.54 -0.16, 1.23)
	Without other supplements	0.04 (-1.32, 1.41)	-	-0.23 (-1.73, 1.26)	-0.09 (-1.77, 1.59)
**Basal covariates**					
Stojanovié M et al (2017)	FPG: < 140 mg/dL	-1.40 (-6.80, 3.80)	-	-	-
	FPG: > 140 mg/dL	-12.80 (-22.30, -3.10)	-	-	-
	FPG: < 108 mg/dL	0.00 (-5.20, 5.00)	-	-	-
	FPG: > 108 mg/dL	-11.70 (-19.30, -4.00)	-	-	-
**High quality**					
Kim Y et al (2022)	-	-8.84 (-16.94, -0.75)	-0.23 (-0.40, -0.05)	-	-0.83 (-2.12, 0.47)

Table S4. Risk of bias assessment.

Reference	1	2	3	4	5	6	7	8	9	10	11	12	13	14	15	16	Overall
Suksomboon N et al (2015)	YES	NO	YES	PY	YES	YES	YES	YES	YES	NO	YES	NO	NO	NO	YES	YES	**Critically low**

Sahebkar A et al (2016)	YES	NO	YES	PY	NO	NO	NO	YES	YES	NO	YES	NO	YES	YES	YES	NO	**Critically low**
Moradi M et al (2016)	YES	NO	YES	PY	NO	YES	NO	YES	NO	NO	YES	NO	NO	YES	YES	YES	**Critically low**
Stojanović M et al (2017)	YES	NO	YES	PY	NO	NO	NO	YES	YES	NO	YES	YES	YES	YES	YES	YES	**Critically low**
Jorat MV et al (2018)	YES	NO	YES	PY	YES	YES	NO	YES	YES	NO	YES	NO	YES	YES	YES	YES	**Critically low**
Shi-Ying Z et al (2018)	YES	YES	YES	PY	YES	YES	NO	YES	YES	NO	YES	YES	YES	YES	YES	YES	**Low**
Sharifi N et al (2018)	YES	YES	YES	PY	YES	YES	YES	YES	YES	YES	YES	YES	YES	YES	YES	YES	**High**
Bakhshayeshkaram M et al (2018)	YES	NO	YES	PY	YES	YES	NO	YES	YES	NO	YES	NO	YES	YES	YES	YES	**Critically low**
Zhang X et al (2019)	YES	NO	YES	PY	YES	YES	NO	YES	YES	NO	YES	YES	YES	YES	YES	YES	**Critically low**
Dludla PV et al (2020)	YES	NO	YES	PY	YES	YES	YES	YES	YES	NO	YES	NO	YES	YES	YES	YES	**Low**
Xu Y et al (2021)	YES	YES	YES	PY	YES	YES	NO	YES	YES	NO	YES	YES	NO	NO	YES	YES	**Critically low**
Zhang J et al (2021)	YES	YES	YES	PY	YES	YES	NO	YES	YES	NO	YES	YES	YES	YES	YES	YES	**Low**
Liang Y et al (2022)	YES	YES	YES	PY	YES	YES	NO	YES	YES	YES	YES	YES	YES	YES	YES	YES	**Low**
Zhang T et al (2022)	YES	YES	YES	PY	YES	YES	YES	YES	YES	NO	YES	YES	YES	YES	NO	YES	**Low**
Kim Y et al(2022)	YES	YES	YES	PY	YES	YES	YES	YES	YES	YES	YES	YES	YES	YES	YES	YES	**High**
Liu Z et al (2022)	YES	YES	YES	PY	YES	YES	NO	YES	YES	YES	YES	NO	YES	YES	YES	YES	**Low**
Ardekani A et al (2023)	YES	NO	YES	PY	YES	YES	NO	YES	YES	NO	YES	NO	YES	YES	YES	YES	**Critically low**

Table S5. Quality of evidence assessment.

Assessment							Quality
**Nº studies**	**Design**	**RoB**	**Inconsistence**	**Indirect evidence**	**Imprecision**	**Others**	
**A. Total Cholesterol**							
**13**	RCT	Very serious	Not serious	Not serious	Serious	None	Very low
**B. LDL-C**							
**13**	RCT	Very serious	Not serious	Not serious	No seria	None	Low
**C. HDL-C**							
**13**	RCT	Very serious	Not serious	Not serious	Serious	None	Very low
**D. Triglycerides**							
**13**	RCT	Very serious	Not serious	Not serious	Serious	None	Very low
**E. Lp(a)**							
**2**	RCT	Serious	Serious	Not serious	Very serious	None	Very low
**F. FPG**							
**12**	RCT	Serious	Not serious	Not serious	Serious	Strong association	Moderate
**G. HbA1c**							
**9**	RCT	Serious	Not serious	Not serious	Not serious	Strong association	High
**H. Fasting insulin**							
**8**	RCT	Serious	Not serious	Not serious	Serious	None	Low
**I. HOMA-IR**							
**7**	RCT	Serious	Not serious	Not serious	Very serious	None	Very low

Figure S1. Effect of CoQ10 supplementation on Lp(a).

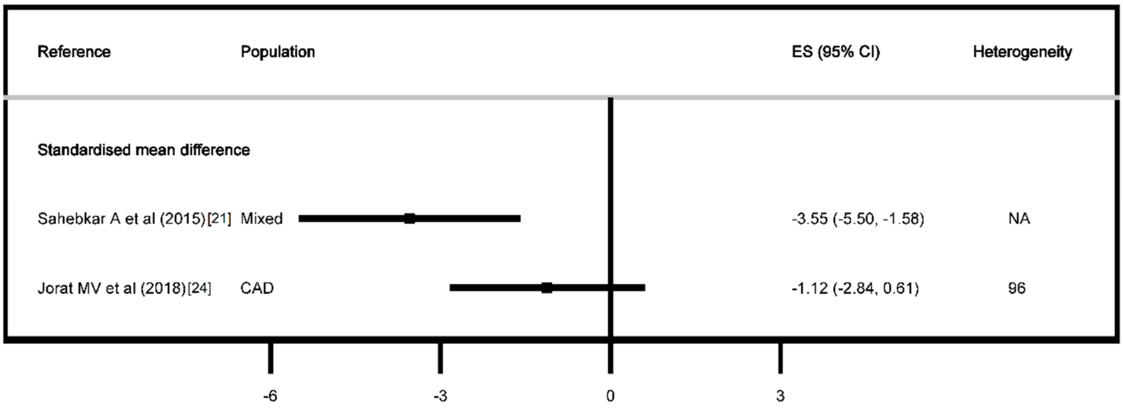

Appendix S1. Search strategy.

- Medline, Scopus, Web of Science, and Cochrane Library

("coenzyme q10" OR q10 OR ubiquinol OR ubiquinone) AND (dyslipidemia OR "lipidic profile" OR cholesterol OR hdl OR "High-density lipoprotein" OR ldl OR "low-density lipoprotein" OR triglycerides OR "lipoprotein A" OR "fasting blood glucose" OR "basal blood glucose" OR "basal glycemia" OR "fasting glucose" OR "glycated hemoglobin" OR a1c OR "fasting insulin" OR "homa-ir") AND ("systematic review" OR "meta-analysis")

- Grey literature

Not specified.
